# A Human TREK-1/HEK Cell Line: A Highly Efficient Screening Tool for Drug Development in Neurological Diseases

**DOI:** 10.1371/journal.pone.0025602

**Published:** 2011-10-14

**Authors:** Hamid Moha ou Maati, Rémi Peyronnet, Christelle Devader, Julie Veyssiere, Fabien Labbal, Carine Gandin, Jean Mazella, Catherine Heurteaux, Marc Borsotto

**Affiliations:** Institut de Pharmacologie Moléculaire et Cellulaire, Centre National de la Recherche Scientifique (CNRS, UMR6097), Université de Nice Sophia Antipolis, Valbonne, France; University of Muenster, Germany

## Abstract

TREK-1 potassium channels are involved in a number of physiopathological processes such as neuroprotection, pain and depression. Molecules able to open or to block these channels can be clinically important. Having a cell model for screening such molecules is of particular interest. Here, we describe the development of the first available cell line that constituvely expresses the TREK-1 channel. The TREK-1 channel expressed by the h-TREK-1/HEK cell line has conserved all its modulation properties. It is opened by stretch, pH, polyunsaturated fatty acids and by the neuroprotective molecule, riluzole and it is blocked by spadin or fluoxetine. We also demonstrate that the h-TREK-1/HEK cell line is protected against ischemia by using the oxygen-glucose deprivation model.

## Introduction

Potassium (K^+^) channels are important in brain and cardiovascular diseases both as drug targets and as a cause of underlying pathology. In mammalian excitable cells, their opening results in an inhibitory hyperpolarization, while their closing induces an excitatory depolarization. The therapeutic potential of K^+^ channels as drug targets in medicine is widely recognized, and is an area of active ongoing research. To date, the class III antiarrhythmics, the sulfonylureas used in diabetes mellitus are K^+^ channel inhibitors, and some vasodilators such as nicorandil are K^+^ channel openers.

Among the large K^+^ channel class, the most recently identified family, is that of the two-pore-domain K^+^ channels (K2P) with four transmembrane segments and two pore-forming P loops [1]. These channels set the membrane potential towards the K^+^ equilibrium potential [1], [2]. They can be found in excitable or in non-excitable cells. Many K2P are polymodal and respond to a wide range of different regulatory inputs. Rapidly, several K2P channels were identified to be involved in a great diversity of functional roles and responsible for important diseases. TASK-1 (KCNK-3) plays an important role in potassium dependent apoptosis [3] and in central nervous system inflammation [4]. Mutations in the gene of TASK-3 (KCNK9) cause a syndrome of mental retardation [5]. Double TASK-1/TASK-3 deletion in mice also causes primary hyperaldosteronism [6]. TRESK-1 (KCNK18) was recently shown to be involved in pain and migraine [7]. TRAAK (KCNK4) alone or in association with TREK-1 (KCNK2) controls thermal responses of both capsaicin-sensitive and capsaicin-insensitive sensory neurons [8], [9].

The TREK-1 channel is the most extensively studied of the K2P channels [1], [2]. This stretch activated channel is widely expressed in the brain where it is abundant in regions such as putamen, caudate nucleus, prefrontal cortex, hypothalamus, hippocampus and dorsal raphe, cerebral structures strongly involved in depression [10]-[12]. TREK-1 is also present in the dorsal root ganglia (DRG) where it is colocalized with transient receptor potential (TRP) channels, that are involved in thermosensation [8], [9]. The TREK-1 channel is also expressed in peripheral tissues like smooth muscles of the gastrointestinal tract or the prostate [11], [12], [13]. In the cardiovascular system, expression of this channel has been reported in mesenteric, pulmonary and basilar arteries [14], [15] as well as in skin microvessels [16]. In the heart, it has been identified in rat left and right ventricles, atria and septum, and in ventricular myocytes [17], [18] but not in the human myocardium. Pharmacologically, TREK-1 is insensitive to all the “classical” K^+^ channel blockers such as 4-AP (4-aminopyridine) or TEA (triethylammonium). Importantly, the complex gating properties of TREK-1 and its modulation by numerous chemical and physical physiological stimuli suits it well to a role in regulating the membrane potential and excitability in various cell types under a range of physiological and pathological situations. This channel is opened by intracellular acidosis, increasing temperatures, phospholipids, the neuroprotective riluzole, volatile and gaseous anesthetics and membrane stretch [19]–[24]. TREK-1 is blocked by the stimulation of both Gq- and Gs-coupled membrane receptors. Interestingly, antidepressant selective serotonin reuptake inhibitors (SSRIs) induce a potent inhibition of this channel [25], [26]. These particular ways of TREK-1 modulation together with the generation of knock-out mice for TREK-1 (TREK-1^−/−^) has allowed the demonstration that TREK-1 channels play a key role in the cellular mechanisms of anaesthesia ^18^, neuroprotection [19], [27], pain [8], [9] and depression [25], [28]. TREK-1^−/−^ mice are less sensitive to volatile anesthetic such as chloroform, halothane, isoflurane or desflurane than wild type mice. Gaseous anesthetics such as xenon, nitrous oxide and cyclopropane activate TREK-1 [24]. Polyunsaturated fatty acids (PUFAs) such as Arachidonic acid (AA), alpha-linolenic acid (ALA), docosahexaenoic acid (DHA) or lysophospholipids (LP) such as lysophosphatidylcholine (LPC) also activate TREK-1 channels and are neuroprotective against both ischemia and seizures induced by kainate injections [22], [29]. In mice, the deletion of the kcnk2 gene results in an increased sensitivity to both ischemia and epilepsy [22]. TREK-1 channels can be opened by painful stimulations like pressure or heat. TREK-1^−/−^ mice are more sensitive than wild type mice to pain induced by heat or mechanical stimuli [8]. TREK-1 channels were reported to be very important in mood regulation. In five behavioral tests TREK-1^−/−^ mice display a depression resistant phenotype [25]. Thus, it has been hypothesized that a TREK-1 blocker could be a potent antidepressant molecule. This hypothesis was validated by the discovery of spadin, that is a sortilin derived peptide and that specifically blocks TREK-1 channels [28], [30]. Mice treated with spadin behave similarly to TREK-1^−/−^ or fluoxetine-treated mice. Consequently, TREK-1 channels have become very attractive molecular and pharmacological targets for the development of new molecules with neuroprotective effects or the design of new drugs for treating pain or mood disorders like depression. Until now, there are no available cell lines to easily screen such molecules. TREK-1 channels have to be transfected into regular cell lines like COS-7, CHO and HEK293. For rapid and efficient screenings, these techniques raise different problems such as the yield of transfection and the level of expression. To avoid these difficulties we developed a HEK293 clone, that we called h-TREK-1/HEK which stably expresses the TREK-1 channel. When compared to the native channel, the TREK-1 channel expressed in the h-TREK-1/HEK clone conserves the sensitivity to its known activators such as stretch, polyunsaturated fatty acids, internal pH decrease and riluzole, and to its blockers such as fluoxetine and spadin. Because hypoxia plays a key role during ischemia, we highlighted the potent role of the TREK-1 channel in the protection against ischemia in the h-TREK-1/HEK cell line.

## Results

### h-TREK-1/HEK cell line

**Figure 1:**
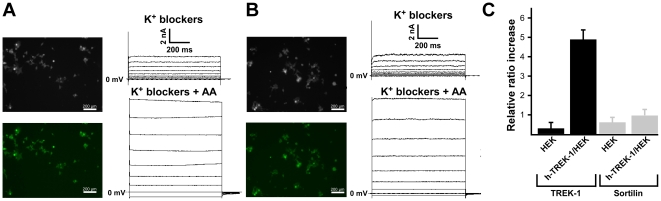
GFP expression and functional h-TREK-1 channel activity on h-TREK-1/HEK cells. **A**) Typical pictures of cells observed in transmission and in fluorescence at round three of cell culture. Functional channel activity was evaluated by current activation with 10 µM AA. **B**) Typical pictures of cells observed in transmission and in fluorescence at round fourteenth of cell culture. Functional channel activity was evaluated by current activation with 10 µM AA. **C**) Real time q-PCR. Levels of TREK-1 or sortlin expression were normalized with the cyclophillin D expression.

A vector expressing h-TREK-1 (pIRES2-eGFP) was transfected into HEK-293 cells in the presence of 1.5 mg/ml Geneticin (G418). After three or four weeks of culture, G418 resistant clones were isolated and reseeded in the same G418 medium (0.5 mg/mL instead of 1.5 mg/ml). At this stage, all clones were electrophysiologically tested after 48 h of culture and only clones where each single cell expressed the TREK-1 channel were retained (positive clone). Each positive clone was checked after each round of culture. After 14 rounds of culture, we did not observe variations either in the TREK-1 current expression or in the EGFP expression (Figure 1A,B). The TREK-1 channel expression was also measured by q-PCR. Results revealed a large increase in the TREK-1 mRNA expression in the h-TREK-1/HEK cells (Figure 1C).

To validate the selected clone we studied the modulation of the TREK-1 channel activity by applying variations of membrane stretch or internal pH values, current activators (PUFAs, riluzole) or specific blockers of TREK-1(spadin, fluoxetine). We also confirmed the ability of TREK-1 to bind ^125^I-spadin.

### Mechanoactivation

**Figure 2:**
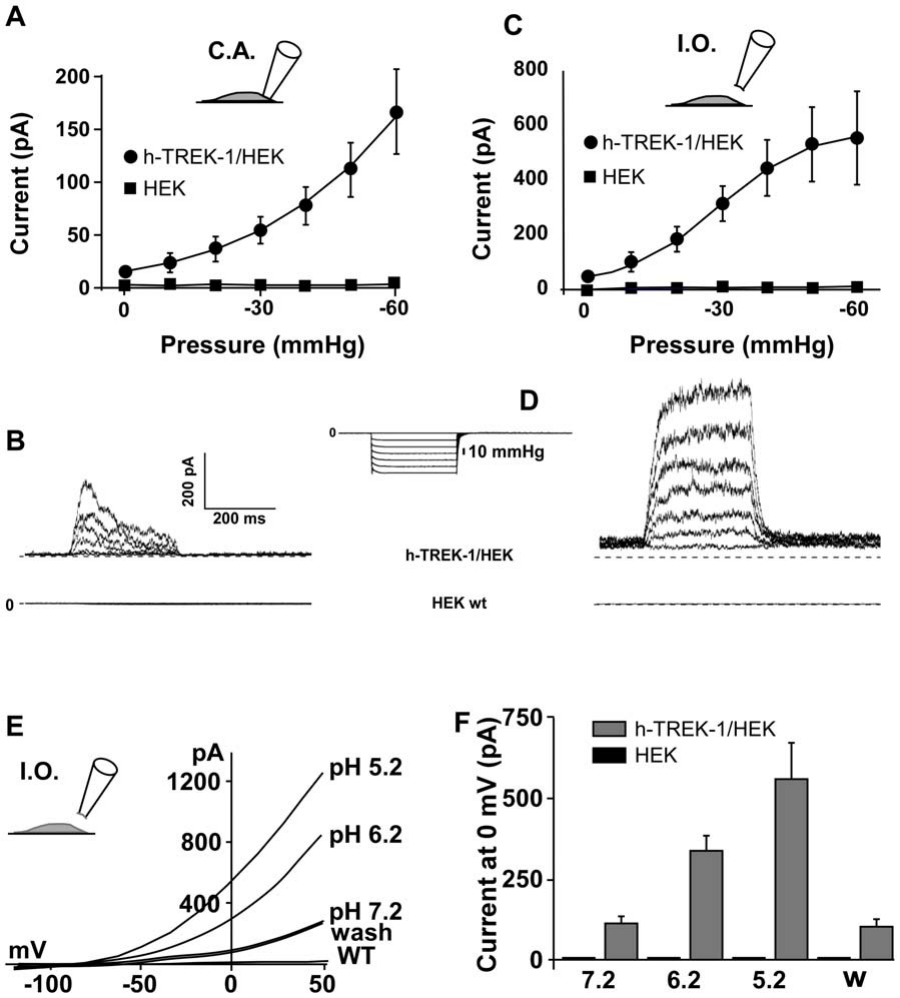
Stretch and pH activation of h-TREK-1 current in cell attached (C.A.) and in inside out (I.O.) patch clamp configurations. Currents were recorded by application of negative pressure and by internal acidification. **A, B**) Current/pressure relationship and typical current traces obtained on h-TREK-1/HEK cells (closed circle, n = 30) and on HEK cells (closed square, n = 5) in cell-attached patch clamp configuration. **C, D**) Current/pressure relationship and typical current traces obtained on h-TREK-1/HEK cells (closed circle, n = 30) and on HEK cells (closed square, n = 4) in inside-out patch clamp configuration. Inset: negative pressure step protocol, increase −10 mmHg. **E, F**) Current/potential curves and corresponding histograms obtained after internal acidification on h-TREK-1/HEK cells (n = 8) and on HEK cells (n = 3).

Patches were stimulated with negative pressure pulses, from 0 to −60 mmHg in −10 mmHg increment. No current increases were observed in native HEK cells either in the cell attached (C.A.) (Figure 2A) or in the Inside-Out (I.O.) (Figure 2C) patch configurations. With the h-TREK-1/HEK clone in C.A. mode the current regularly increased with a hyperbolic pattern from 14.5±1.5 pA to 167.14±41.8 pA when the pressure decreased from 0 mm Hg to −60 mm Hg (Figure 2A, B). In the I. O. mode we observed a sigmoïdal increase with a plateau value around −50 mm Hg. The maximum current was 542.7±161.9 pA at −60 mm Hg (Figure 2C,D). Differences in current values were expected because in the C.A. mode the cytoskeleton induced an inhibitory effect on TREK-1 current. The cytoskeleton effect disappeared in the I.O. mode. These differences also explained the differences observed in the current patterns (Figure 2B and Figure 2D).

### Sensitivity to internal pH

Lowering the internal pH changes the mechanosensitive TREK-1 channel into a constitutive leak channel [31]. In the I.O. patch configuration from h-TREK-1/HEK clone, the TREK-1 current increased with the acidification from pH 7.2 to pH 5.2. Current values at 0 mV were 81.6±18.1 pA, 294.8±51.6 pA and 543.2±116.7 pA, at pH values of 7.2, 6.2, and 5.2, respectively (Figure 2E,F). At the same pH values, currents of HEK were 12.3±0.4 pA, 12.8±0.2 pA and 12.9±0.3 pA, respectively (Figure 2F). These values were reversible because after a wash with the bath solution the current value returned to the value measured at pH 7.2, 79.2±14.4 pA and 11.9±0.7 pA for h-TREK-1/HEK and HEK, respectively (Figure 2E,F).

All the measurements of the following experiments described below were performed in the presence of a cocktail of potassium channel inhibitors which did not affect TREK-1 channel activity.

### Activation of TREK-1 by polyunsaturated fatty acids

**Figure 3:**
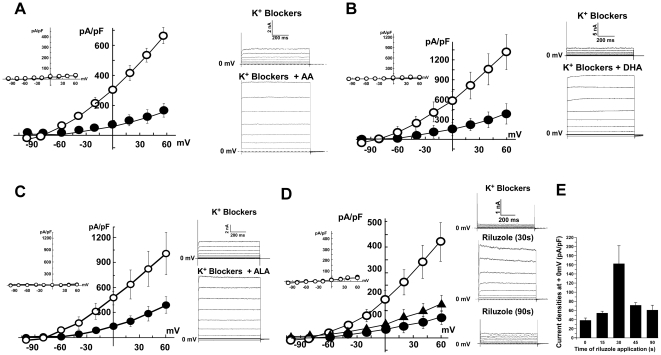
Pharmacological activation of h-TREK-1 current in whole cell patch clamp configuration. Currents were recorded in the presence of a cocktail of potassium channel inhibitors (K^+^ blockers). **A**) Current/potential curves and corresponding current traces obtained before (closed circle) and after (open circle) current activation by AA 10 µM (n = 118). **B**) Current/potential curves and corresponding current traces obtained before (closed circle) and after (open circle) current activation by DHA 10 µM (n = 10). **C**) Current/potential curves and corresponding current traces obtained before (closed circle) and after (open circle) current activation by ALA 10 µM (n = 10). **D**) Current/potential curves and corresponding current traces obtained before (closed circle) and after current activation by riluzole 100 µM (n = 12) perfused during 30 s (open circle) or 90 s (closed triangle). Each pharmacological activator was tested on HEK-293 native cells (n = 10) and current/potential curves were shown in the inset of each curve. **E**) Current density values measured at 0 mV after different times of perfusion of 100 µM riluzole (n = 10 at each time value).

Polyunsaturated fatty acids (PUFAs) like arachidonic acid (AA), docosahexaenoic acid (DHA) or alpha- linolenic acid (ALA) have the ability to reversibly activate the TREK-1 channel. These effects have been demonstrated to be direct on the channel [32]. Thus, we measured their effects on the TREK-1 channel expressed by the h-TREK-1/HEK cell line. The three PUFAs were able to largely increase the TREK-1 activity. Current densities measured at 0 mV in the presence of K^+^ blockers or in the presence of K^+^ blockers + PUFA were 60±5.9 pA/pF versus 306.2±21.7, 159.9±42.0 pA/pF versus 583.7±113.1 pA/pF and 131.8±25.0 pApF versus 467.0±114.9 pA/pF for AA, DHA and ALA, respectively (Figure 3A–C).

### Activation of TREK-1 by the neuroprotective molecule riluzole

Riluzole is an important neuroprotective molecule with anticonvulsant and anti-ischemic properties [23], [33]–[35]. Riluzole has been described to induce a transient activation measured 30 s after drug application followed by an inhibition measured at 90 s [33]. These biphasic effects were also present with the TREK-1 channel expressed by the h-TREK-1/HEK cell line (Figure 3D,E). Current density values were of 37.4±6.4 pA/pF in the presence of K^+^ blocker cocktail alone and were increased to 161.8±40.2 pA/pF after 30 s in the presence of 100 µM riluzole and returned to 60.1±11.6 pA/pF after 90 s (Figure 3D,E).

### Inhibition of TREK-1 by spadin and fluoxetine

**Figure 4:**
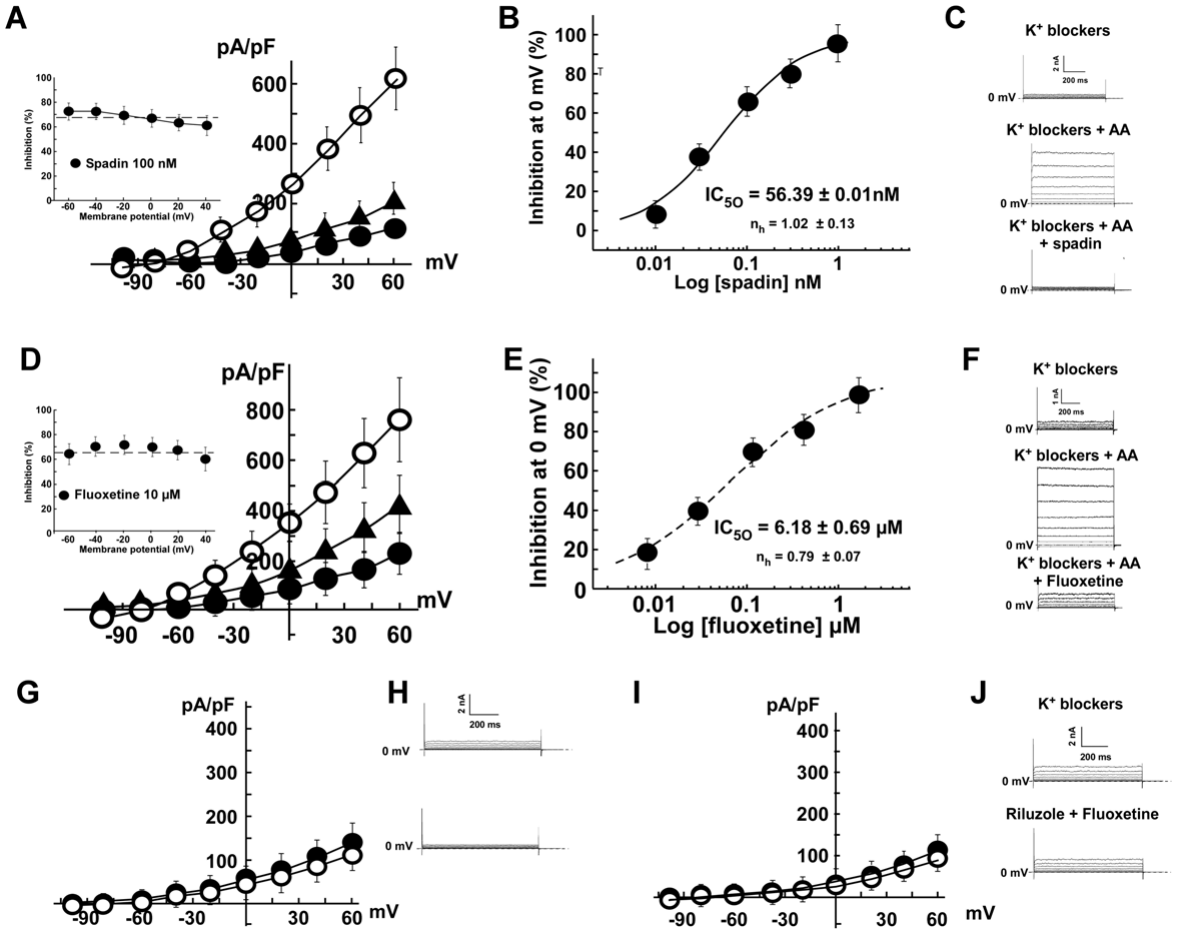
Pharmacological inhibition of h-TREK-1 current in whole cell patch clamp configuration by spadin (n = 12 for each dose) and fluoxetine (n = 12 for each dose). Currents were recorded in the presence of a cocktail of potassium channel inhibitors (K^+^ blockers). The inhibition was obtained after the pre-activation of the current by 10 µM AA. **A)** Current/potential curves obtained in presence of K^+^ blockers (closed circles), K^+^ blockers + AA (open circles) and K^+^ blockers + AA + spadin 1 µM (closed triangles). The absence of voltage dependence of spadin inhibition (100 nM) was shown in the inset. **B)** Spadin dose dependent inhibition at 0 mV potential. **C)** Typical traces of hTREK-1 current pre-activated by 10 µM AA and inhibited by 1 µM spadin. **D)** Current/potential curves obtained in the presence of K^+^ blockers (closed circles), K^+^ blockers + 10 µM AA (open circles) and K^+^ blockers + 10 µM AA + 30 µM fluoxetine (closed triangles). The absence of voltage dependence of fluoxetine inhibition (10 µM) was shown in the inset. **E)** Fluoxetine dose-dependent inhibition at 0 mV potential. **F)** Typical traces of h-TREK-1 current pre-activated by 10 µM AA and inhibited by 30 µM fluoxetine. **G, H)** Current/potential curves and representative traces of spadin inhibition (1 µM) on 100 µM riluzole activated hTREK-1 current (n = 10). **I, J)** Current/potential curves and representative traces of fluoxetine inhibition (30 µM) on 100 µM riluzole activated hTREK-1 current (n = 10).

TREK-1 channels play a key role in mechanisms of depression [25]. Recently, we demonstrated that TREK-1 channels are inhibited by spadin, a peptide that represents a new concept in antidepressant drug design [28]. Here, we showed that spadin blocked TREK-1 current exactly as it did for the transiently transfected TREK-1 channels (Figure 4A–C). For example, 100 nM spadin inhibited 70% of the TREK-1 current measured at 0 mV (Figure 4A). The membrane potential did not significantly affect the ability of spadin to inhibit the TREK-1 channel (Figure 4A inset). The dose for inhibiting 50% of the TREK-1 channel activity (IC_50_) was 56.39±0.01 nM (Figure 4B). It was previously shown that TREK-1 channels can be directly blocked by antidepressant drugs such as Serotonin Selective Reuptake Inhibitors (SSRIs) like fluoxetine [26]. TREK-1 channels expressed by the h-TREK-1/HEK cell line were inhibited by fluoxetine (Figure 4D–F). 10 µM of fluoxetine inhibited 67% of the TREK-1 current measured at 0 mV (Figure 4D). Here again, the membrane potential did not alter the inhibiting ability of fluoxetine (Figure 4D inset). The IC_50_ for fluoxetine was 6.18±0.69 µM (Figure 4E). Additionally, spadin and fluoxetine prevented the channel activation by riluzole (Figure 4G–J). Current density values were 40.5±9.8 pA/pF in the presence of the K^+^ blocker cocktail alone and were not significantly modified by 100 µM of riluzole in the presence of 100 nM of spadin, 49.7±11.8 pA/pF (Figure 4G,H). The same pattern was observed with fluoxetine, 27.4±10.3 pA/pF in the presence of the K^+^ blocker cocktail alone and 33.5±6.5 pA/pF in the presence of 100 µM of riluzole and 10 µM of fluoxetine (Figure 4I,J).

### Binding experiments

**Figure 5:**
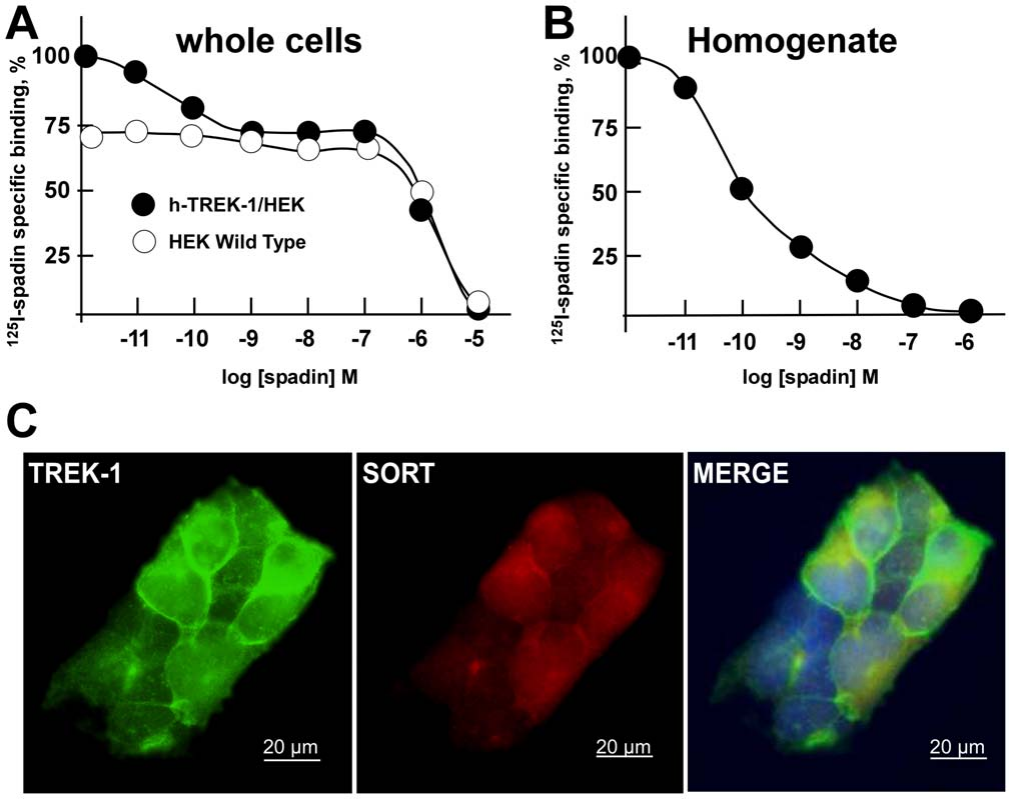
^125^I-spadin competition and immunolabeling experiments on h-TREK-1/HEK cells and HEK cells. **A, B)** ^125^I-spadin competition experiments performed on whole cells (n = 3) and on plasma membrane homogenate (n = 3). **c)** Immunolabelling of h-TREK-1 (green labeling) and sortilin (red labeling) proteins. The co-localization of the two proteins is shown on the Merge picture.

We previously determined that iodinated spadin was able to bind on TREK-1 channels [28]. In order to distinguish the contribution of TREK-1 channels and sortilin for the total binding, we performed competition experiments on intact cells (TREK-1 and sortilin) and plasma membrane preparations (homogenate, only TREK-1) from h-TREK-1/HEK cells. As previously reported [28], we observed that there was two binding sites on whole cells with apparent K_0.5_ of 10^−6^ M and 10^−10^ M (Figure 5A) The low affinity binding sites were also present on HEK cells (Figure 5A) and disappeared in homogenate preparations indicating that they correspond to the sortilin receptor. The high affinity binding sites, that were absent in HEK wild type cells, were due to the presence of TREK-1 channels (Figure 5B).

### Colocalization between TREK-1 channel and Sortilin receptor

HEK cells constitutively expressed the sortilin receptor which was not increased by the stable expression of h-TREK-1 channels (Figure 1C). We previously determined that the sortilin receptor is an addressing partner to the plasma membrane for TREK-1 channels [28]. As expected, we showed by immunolabelling that both proteins were co-localized at the plasma membrane level in the h-TREK-1/HEK cell line (Figure 5C).

### Protective effects of TREK-1 channel against hypoxia

**Figure 6:**
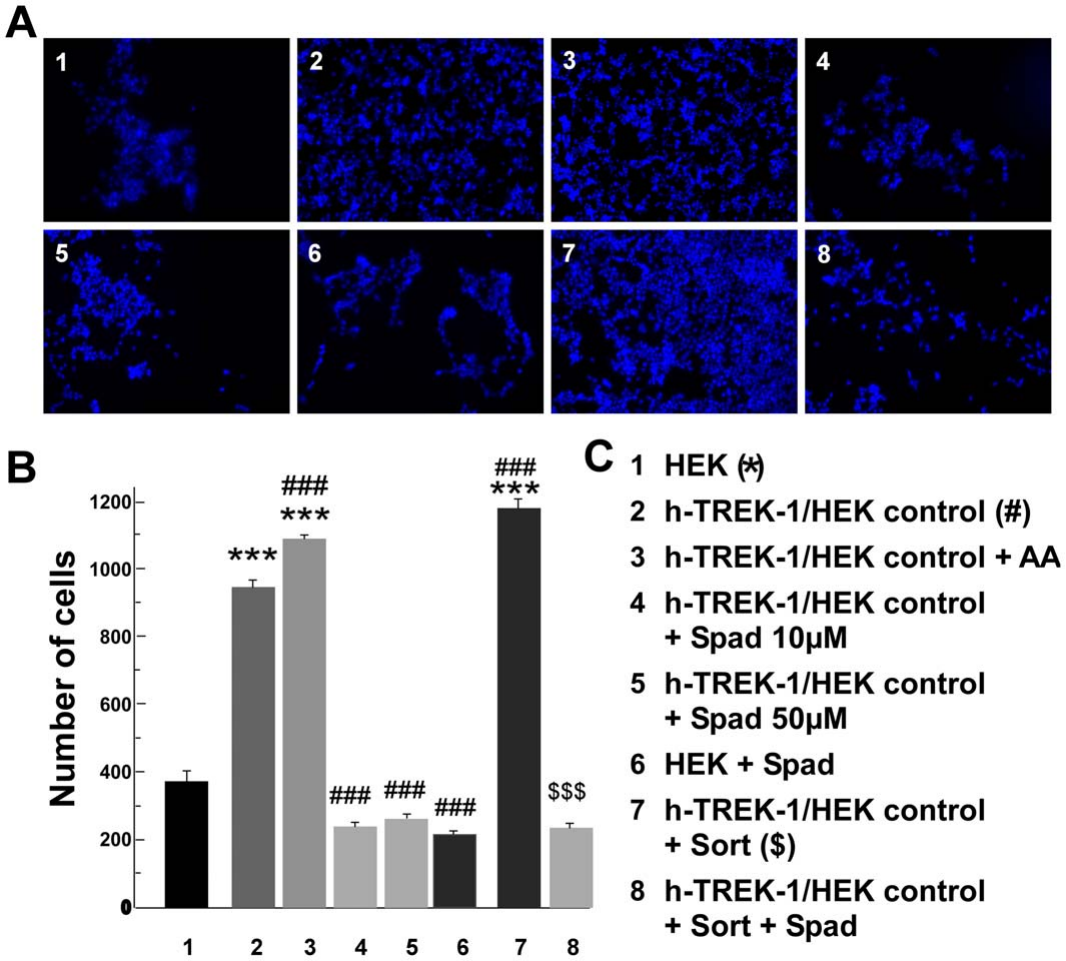
Oxygen deprivation glucose experiments (OGD) on h-TREK-1/HEK cells and on HEK cells (A, B). After two hours of OGD in control conditions or with different treatments, survival cells were fixed, labeled and counted (n = 8 for each groups). **A)** Typical pictures of cells from each tested condition. **B)** Histograms of number of survival cells (Tukey test, F_7,568_ = 38.65; *** or ### or $$$ p<0.001) **c)** The different tested conditions and their corresponding number.

We studied the protective role of TREK-1 channels by using the OGD protocol, which is considered as the best reliable *in vitro* model of ischemia [36], [37]. OGD consists in a glucose and oxygen deprivation, 1.2% instead of 5% in normal conditions. HEK and h-TREK-1/HEK cells were grown in different conditions and, after two hours of OGD, the number of cells which survived was counted in 9 areas of the Petri dish, these 9 areas were selected randomly by a computer. It clearly appeared that TREK-1 expression increased the survival, 934±17 versus 383±19 for h-TREK-1/HEK and HEK cells, respectively (Figure 6, conditions 1 and 2). Application of 10 µM AA, an activator of TREK-1 induced an increase of cell survival (Figure 6, condition 3). For the h-TREK-1/HEK cell line treated with 10 or 50 µM spadin the number of survival cells is close to that of HEK treated with 10 µM spadin (Figure 6, conditions 4, 5 and 6). The expression of the sortilin receptor, which increased the number of TREK-1 channels at the plasma membrane induced a higher increase in the survival (1124±15 compared to 934±17: conditions 7 and 2). This sortilin-induced increase was prevented by a 10 µM spadin treatment (Figure 6, condition 8). All these results showed that the presence of TREK-1 in the stable HEK cell line is protective against ischemia and that spadin, the specific inhibitor of TREK-1 potently reverses this effect.

## Discussion

The generation of TREK-1 knock-out mice (KCNK2^−/−^) has enabled direct evaluation of the contribution of these channels to native currents. More importantly, this mouse strain has been used to examine proposed roles for TREK-1 channels in physiological functions or drug action. Because, there are no tissues or cell lines that express TREK-1 at a sufficient level, all studies concerning the TREK-1 channel have been conducted in transient transfected cells. This technique poses several problems including the number of cells that are transfected, the level of expression of the channel in the transfected cells and the time during which the cells are available for measurements. These problems render difficult and longer large scale screenings of compounds interacting with the TREK-1 channel. Here, we developed a cell line, the h-TREK-1/HEK that allows avoiding all these problems. At least up to 14 rounds of culture, each single cell expresses the channel at a similar level and the culture can be still maintained for several days or weeks. The generation of the h-TREK-1/HEK cell line, which stably expresses a functional h-TREK-1 channel with all its regulation pathways represents a highly efficient screening method for the development of drugs modulating the TREK-1 channel. This is of particular importance because TREK-1 channels are involved in several physiopathological processes such as pain [8], [9], [38], neuroprotection [19], [22], [23], [33] and mood disorders [25], [28]. Thus, molecules or drugs able to activate TREK-1 could be potentially used for treating pain or as neuroprotective molecules against stroke or epilepsy. Conversely, molecules or drugs able to inhibit this channel will be useful for treating depression. Thanks to the use of the h-TREK-1/HEK cell line, TREK-1 modulators (openers and blockers) will be now screened more rapidly and more efficiently. Generally, cell lines that stably express ionic channels are currently used for pharmacological studies such as HERG and KVLQT1 channels, the two most important repolarizing cardiac currents responsible for long QT syndrome and Torsades de Pointes [39], [40]–[42].

Such a method targeting the TREK-1 channel will potentially allow the discovery and development of new drugs against neurological disorders such as depression, epilepsy and ischemia. It will be also crucial to identify new anesthetics and analgesic molecules. Activation of TREK-1 channels by volatile and gaseous anesthetics [24] may also be important for their anesthetic activity *in vivo*. TREK-1 channels also contribute to temperature and mechanical pain sensation. Single C-fibers recordings in skin-nerve preparations from TREK-1 KO mice revealed heightened sensitivity to raised temperature over a range where TREK-1 channels are strongly activated (30–45°C). Enhanced sensitivity to mechanical stimuli is also observed in TREK-1 deficient mice, while increased pain sensitivity associated with inflammation is reduced in these mice. These results indicate that drugs targeting TREK-1 channels obtained by screening with the h-TREK-1/HEK cell line could be used in pain treatment. TREK-1 activators might increase threshold for thermal and/or mechanical pain, whereas blockers of TREK-1 could decrease hyperalgesia associated with inflammation.

TREK-1 is resistant to hypoxia and opens during chemical ischemia [43], [44]. Polyunsaturated fatty acids, riluzole and lysophospholipids, that are potent openers of TREK-1 [31], [33], [45] protect rodent brain against global, focal and spinal ischemia and induce ischemic and epileptic tolerance [19], [34], [46]–[50]. Ischemia results from an interruption in the arterial blood supply to the brain, resulting in restricted oxygen and glucose supply to tissue distal to the obstruction. During brain ischemia, endogenous AA is released, intracellular pH falls and neurons swell. These alterations contribute to opening of TREK-1 channels leading to a hyperpolarisation that lowers intracellular calcium and limits glutamate excitotoxicity. The crucial role of TREK-1 in PUFA- and LP-induced neuroprotection against ischemia was further indicated by the fact that TREK-1 deficient mice display an increased sensitivity to ischemic insult and that the neuroprotection by PUFAs and LPs disappears in the KO mice [22].*In vitro* models of ischemia, although isolated from the effects of cardiovascular system, aim specifically to mimic the rapid depletion of oxygen and glucose seen under ischemic conditions *in vivo*. Thus, such models of oxygen and glucose deprivation (OGD) allow specific mechanistic processes to be investigated in a drug-naïve environment as well as the screening of potential neuroprotective molecules. In this work, we applied the OGD model on the h-TREK-1/HEK cell line. Clearly, the h-TREK-1/HEK cell line, where all single cells express the TREK-1 channel is protected against the deleterious neuronal damage induced by OGD. Application of AA enhances the neuroprotection induced by the presence of TREK-1 in the cell line, which is prevented by spadin, the specific blocker of TREK-1. All these results demonstrate that TREK-1 channels are resistant to hypoxia. The h-TREK-1/HEK cell line has allowed the first direct demonstration of the involvement of TREK-1 channels in the protection against ischemia. In addition, this work proves that the h-TREK-1/HEK cell line represents a reliable and efficient tool to investigate different strategies of neuroprotection targeting TREK-1. In addition to its function on neuronal activity, it is also known that activation of TREK-1 channels enhances collateral blood flow during cerebral ischemia [14]. TREK-1 channels are necessary for receptor-mediated generation of nitric oxide by vasodilatators such as acetylcholine in endothelial cells [14], [16]. They also contribute directly to hyperpolarizing and relaxing effects of PUFAs on vascular smooth muscle [14], [16]. The h-TREK-1/HEK cell line might be very useful for the screening of new vasodilators. Finally, it was reported that specific TREK-1 antagonists might be useful agents for the treatment of depression, since the deletion of TREK-1 induces a depression-resistant phenotype. The discovery of spadin afforded this hypothesis. Spadin develops antidepressant properties and represents a new concept of antidepressant drug design. Binding experiments and colocalisation between TREK-1 and sortilin in the h-TREK-1/HEK cell line are good arguments for further identification of new TREK-1 partner proteins. This work also shows that the h-TREK-1/HEK cell line responds to spadin by inhibiting the TREK-1 channel and highly suggests that it will be useful to efficiently and rapidly screen analogs of spadin with a better affinity and stability.

In conclusion, the development of an available cell line that allows rapid and reproducible large scale and/or high throughput screenings of molecules interacting with TREK-1 represent a very powerful tool. It will allow the identification of high-affinity openers and blockers of TREK-1 that might prove useful for a range of cardiovascular and neuronal disease states. It is also expected to yield additional insights into regulation and physiological functions of TREK-1.

## Materials and Methods

### Cell culture

The coding sequence of the human TREK-1 (kcnk2) gene (GenBank Acc. No. NM_014217) was cloned into pIRES2-eGFP vector (Invitrogen, Cergy-Pontoise, France). In order to obtain a cell line that stably expresses the TREK-1 channel, the plasmid was transfected (25 ng) into HEK-293 cells (American Type Culture Collection, Manassas, VA, USA) using the Calcium Phosphate method as described by the manufacturer protocol in the presence of 1.5 mg/mL Geneticin (G418) (Invitrogen, Cergy-Pontoise, France). After 20–30 days of culture in G418 selection medium, individual colonies of resistant cells were isolated by using cloning cylinders (Sigma Aldrich, Saint-Quentin Fallavier, France). For all subsequent cultures the human-TREK-1/HEK293 (h-TREK-1/HEK) cells were grown in the presence of 0.5 mg/mL G418 in Dulbecco's modified Eagle's medium supplemented with 10% (v/v) heat inactivated fetal bovine serum containing 1% (v/v) penicillin/streptomycin in an atmosphere of 95% air/5% CO_2_. Functional expression was validated by performing electrophysiological measurements on cells seeded 1–2 days before use on glass coverslips with the same medium but without G418. Native HEK293 (HEK) cells were grown in Dulbecco's modified Eagle's medium containing 1% (v/v) of penicillin/streptomycin and Glutamax (Invitrogen, Cergy- Pontoise, France) and supplemented with 10% (v/v) heat inactivated fetal bovine serum in an atmosphere of 95% air/5% CO_2_.

### Real-Time quantitative RT-PCR analysis

Total RNAs from HEK and h-TREK-1/HEK cells were isolated with the RNA easy mini kit (Qiagen, Les Ulis, France). 5 µg of total RNAs were used for reverse transcription reaction carried out with the Superscript II reverse transcriptase (Invitrogen, Cergy-Pontoise, France) according to the protocol of the supplier. Real-time PCR analysis (SYBR green mastermix plus, Eurogentec, Seraing, Belgium) was performed to estimate the level of expression of TREK-1 and the endogenous reference, cyclophilin D (CycloD). Real-time PCR assays (triplicate for each target gene tested) were performed in 96-well plates on an ABI GenAmp 5700 apparatus. Data were analyzed using the comparative Ct method where the amount of target was normalized to the endogenous reference (User bulletin N°2 Applied Biosystems). Primers used for the different amplicons were as follows: TREK-1 forward TTTTCCTGGTGGTCGTCCTC; TREK-1 reverse GCTGCTCCAATGCCTTGAAC; CycloD forward GGCTCTTGAAATGGACCCTTC; CycloD reverse CAGCCAATGCTTGATCATATTCTT.

### Electophysiology

#### Whole cell current recordings

Electrophysiological experiments were performed on native HEK and h-TREK-1/HEK- cells seeded at a density of 20,000 cells/35-mm dishes 2 days before testing. TREK-1 current was recorded using the whole-cell configuration of the patch-clamp technique. Each current was calculated by using a RK 400 patch clamp amplifier (Axon Instrument, Sunnyvale, CA, USA), low-pass filtered at 3 kHz and digitized at 10 kHz using a 12-bit analog-to-digital converter digidata (1322 series, Axon Instrument, Sunnyvale, CA, USA). Patch clamp pipettes were pulled using vertical puller (PC-10, Narishige, London, UK) from borosilicate glass capillaries and had a resistance of 3–5 MΩ. The bath solution contained (in mM) 150 NaCl, 5 KCl, 3 MgCl_2_, 1 CaCl_2_ and 10 HEPES adjusted to pH 7.4 with NaOH. The pipette solution contained (in mM) 155 KCl, 3 MgCl_2_, 5 EGTA and 10 HEPES adjusted to pH 7.2 with KOH. All experiments were performed at room temperature (21–22°C). TREK-1 currents were measured in the presence of a cocktail of potassium channel inhibitors (K^+^ blockers: 3 mM 4-aminopyridine (4-AP), 10 mM tetraethylamonium (TEA), 10 µM glibenclamide, 100 nM apamin and 50 nM charybdotoxin). Stimulation protocols and data acquisition were carried out using a microcomputer (Dell Pentium) which used commercial software and hardware (pClamp 8.2). Cells were clamped at −80 mV and voltage changes were applied by step of 20 mV (from −100 to +60 mV). Duration of depolarization pulses were 0.825 ms, and the pulse cycling rate was 5 s. TREK-1 current amplitudes were calculated at the end of stimulation pulses. Cells were continuously superfused with a microperfusion system. Electrophysiological characterization of human TREK-1 current was obtained by using two channel inhibitors (spadin and fluoxetine) and four activators (three fatty polyunsaturated acids: arachidonic acid, AA; alpha- linolenic acid, ALA, docosahexaenoic acid, DHA, and one benzothiazole: riluzole). Five concentrations of spadin (0.01; 0.03; 0.1; 0.3 and 1 µM) and fluoxetine (1; 3; 10; 30 and 100 µM), and one concentration of AA, ALA, DHA (10 µM) and riluzole (100 µM) were tested on the TREK-1 current. Current amplitudes were expressed in current densities. Results are expressed as mean ± standard error of the mean (SEM). The calculation of the IC_50_ value of spadin and fluoxetine were obtained from dose response curves fitted with a standard sigmoidal Hill function [(y = Vmax (Xn/kn+ Xn))].

#### Stretch experiments

Stretch experiments were performed in both cell attached an inside out patch clamp configurations. The bath solution contained (in mM) 155 KCl, 3 MgCl_2_, 5 EGTA, and 10 HEPES adjusted to pH 7.2 with KOH. The pipette solution contained (in mM) 150 NaCl, 5 KCl, 2 CaCL_2_ and 10 HEPES adjusted to pH 7.4 with NaOH. Patch pipettes were of about 1.5 MΩ membrane, and patches were stimulated with negative pressure pulses, from 0 to −60 mmHg in −10 mmHg increments during 300 ms each 3 s, through the recording electrode using a pressure-clamp device (ALA High Speed Pressure Clamp-1 system; ALA-scientific).

#### pH experiments

These experiments were only performed in inside-out patch clamp configuration. Ramp protocol was used from −120 mV to +50 mV during 550 ms each 3 s. Holding potential was maintained at −80 mV. For all experiments, currents were filtered at 1 kHz, digitized at 20 kHz, and analyzed with pCLAMP9.2 and ORIGIN 6.0 software.

### Binding assays

For competition experiments, intact cells or homogenates from HEK or h-TREK-1/HEK cells were incubated with 0.2 nM ^125^I-spadin (200,000 cpm in 250 µl) iodinated and purified as previously described [28]. Incubations were performed in 50 mM Tris-HCl, pH 7.4 containing 0.1% BSA in the presence of increasing concentrations of non-radioactive spadin (10^−10^ to 10^−5^M). Incubations were ended by addition of 2 ml of ice-cold binding buffer followed by filtration through cellulose acetate filters (Sartorius, Göttingen, Germany) and washing twice with 2 ml of ice-cold buffer. Radioactivity on filters was counted with a gamma-counter.

### TREK-1/NTSR3/Sortilin colocalization experiments

H-TREK-1/HEK cells were first washed for 5 min in PBS, then fixed with 4% paraformaldehyde in PBS for 20 min at room temperature. Coverslips were washed twice with PBS, cells were permeabilized in 0.3% Tween in PBS for 10 min. After 2 hours in PBS containing 2.5% Horse Serum (HS), cells were labeled with a goat polyclonal anti-NTSR3/Sortilin (1/100) and a rabbit anti-TREK-1 (1/3000) (Santa Cruz,Tebu Bio, Le Perray en Yvelines, France), for 16 h at 4°C in PBS containing 5% HS. Cells were washed three times in PBS, then incubated at room temperature in PBS containing FITC conjugated donkey anti-goat antibody (1/1000) and a Texas Red conjugated donkey anti-rabbit antibody (1/1000) in PBS containing 5% HS for 45 min. After two washes with PBS and one with water, coverslips were mounted on glass slides with mowiol for confocal microscopy examination.

### Oxygen Glucose Deprivation (OGD)

HEK-293 and h-TREK-1/HEK cells were seeded at a density of 40,000 cells/35-mm dish. OGD experiments were performed after 2 days of culture [36], [37]. After three washouts with glucose free Earl's balanced salt solution (BSS), cells were maintained in the same BSS medium, (140 NaCl, 5.4 KCl, 1.2 CaCl_2_, 0.9 MgCl_2_, 0.44 KH_2_PO_4_, 4.17 NaHCO_3_ and 0.34 Na_2_HPO_4_ in mM). Prior to use, BSS was equilibrated with the anaerobic gas mixture (95% CO_2_/3.8% N_2_/1.2 O_2_) by bubbling for 15 min, adjusted to pH 7.4 if necessary, and heated to 37°C. Then, cells were placed for two hours in humidified incubator at 37°C in anaerobic gas conditions. After OGD, cells were washed with phosphate buffered saline solution (PBS, Invitrogen, Cergy- Pontoise, France) and then cells were fixed with paraformaldehyde 4% (PAF) at 4°C. Cells were washed 3 times with PBS. Then, nuclei of living cells were labeled by Hoechst during 10 minutes at 4°C. Cells were washed with PBS (×3) and labeled cells were visualized by using a videomicroscope with a Metafluor software. Cell countings were made automatically by ImageJ software and results were expressed as mean ± SEM. OGD was carried out on native HEK 293 and h-TREK-1/HEK cells with different treatments: 1/HEK 293 cells were treated in control conditions or in the presence of spadin (10 µM), 2/h-TREK-1/HEK cells were treated in control conditions or in the presence of AA (10 µM), spadin (10 µM) or spadin (50 µM). For two groups, h-TREK-1/HEK cells were previously transfected (48 hours before OGD) with 25 ng of pcDNA containing sort gene encoding neurotensin receptor 3 called sortilin. For these two groups, OGD was carried out in control conditions or in the presence of spadin (10 µM).

### Statistics

Data were expressed as mean ± S.E.M. Statistical analysis of differences between groups was performed by using unpaired *t* test or Tukey test. In all analyses, the level of significance was set at *P*<0.05 (*), P<0.01 (**) and P<0.001 (***).

## References

[1] Kim D (2005). Physiology and pharmacology of two-pore domain potassium channels.. Curr Pharm Des.

[2] Honore E (2007). The neuronal background K2P channels: focus on TREK1.. Nat Rev Neurosci.

[3] Lauritzen I, Zanzouri M, Honore E, Duprat F, Ehrengruber MU (2003). K+-dependent cerebellar granule neuron apoptosis. Role of task leak K+ channels.. J Biol Chem.

[4] Bittner S, Meuth SG, Gobel K, Melzer N, Herrmann AM (2009). TASK1 modulates inflammation and neurodegeneration in autoimmune inflammation of the central nervous system.. Brain.

[5] Barel O, Shalev SA, Ofir R, Cohen A, Zlotogora J (2008). Maternally inherited Birk Barel mental retardation dysmorphism syndrome caused by a mutation in the genomically imprinted potassium channel KCNK9.. Am J Hum Genet.

[6] Davies LA, Hu C, Guagliardo NA, Sen N, Chen X (2008). TASK channel deletion in mice causes primary hyperaldosteronism.. Proc Natl Acad Sci U S A.

[7] Lafreniere RG, Cader MZ, Poulin JF, Andres-Enguix I, Simoneau M (2010). A dominant-negative mutation in the TRESK potassium channel is linked to familial migraine with aura.. Nat Med.

[8] Alloui A, Zimmermann K, Mamet J, Duprat F, Noel J (2006). TREK-1, a K+ channel involved in polymodal pain perception.. EMBO J.

[9] Noel J, Zimmermann K, Busserolles J, Deval E, Alloui A (2009). The mechano-activated K+ channels TRAAK and TREK-1 control both warm and cold perception.. EMBO J.

[10] Hervieu GJ, Cluderay JE, Gray CW, Green PJ, Ranson JL (2001). Distribution and expression of TREK-1, a two-pore-domain potassium channel, in the adult rat CNS.. Neuroscience.

[11] Medhurst AD, Rennie G, Chapman CG, Meadows H, Duckworth MD (2001). Distribution analysis of human two pore domain potassium channels in tissues of the central nervous system and periphery.. Brain Res Mol Brain Res.

[12] Talley EM, Solorzano G, Lei Q, Kim D, Bayliss DA (2001). Cns distribution of members of the two-pore-domain (KCNK) potassium channel family.. J Neurosci.

[13] Fink M, Duprat F, Lesage F, Reyes R, Romey G (1996). Cloning, functional expression and brain localization of a novel unconventional outward rectifier K+ channel.. EMBO J.

[14] Blondeau N, Petrault O, Manta S, Giordanengo V, Gounon P (2007). Polyunsaturated fatty acids are cerebral vasodilators via the TREK-1 potassium channel.. Circ Res.

[15] Gardener MJ, Johnson IT, Burnham MP, Edwards G, Heagerty AM (2004). Functional evidence of a role for two-pore domain potassium channels in rat mesenteric and pulmonary arteries.. Br J Pharmacol.

[16] Garry A, Fromy B, Blondeau N, Henrion D, Brau F (2007). Altered acetylcholine, bradykinin and cutaneous pressure-induced vasodilation in mice lacking the TREK1 potassium channel: the endothelial link.. EMBO Rep.

[17] Tan JH, Liu W, Saint DA (2004). Differential expression of the mechanosensitive potassium channel TREK-1 in epicardial and endocardial myocytes in rat ventricle.. Exp Physiol.

[18] Terrenoire C, Lauritzen I, Lesage F, Romey G, Lazdunski M (2001). A TREK-1-like potassium channel in atrial cells inhibited by beta-adrenergic stimulation and activated by volatile anesthetics.. Circ Res.

[19] Lauritzen I, Blondeau N, Heurteaux C, Widmann C, Romey G (2000). Polyunsaturated fatty acids are potent neuroprotectors.. Embo J.

[20] Maingret F, Lauritzen I, Patel AJ, Heurteaux C, Reyes R (2000). TREK-1 is a heat-activated background K(^+^) channel.. EMBO J.

[21] Maingret F, Patel AJ, Lesage F, Lazdunski M, Honore E (2000). Lysophospholipids open the two-pore domain mechano-gated K(+) channels TREK-1 and TRAAK.. J Biol Chem.

[22] Heurteaux C, Guy N, Laigle C, Blondeau N, Duprat F (2004). TREK-1, a K(+) channel involved in neuroprotection and general anesthesia.. Embo J.

[23] Heurteaux C, Laigle C, Blondeau N, Jarretou G, Lazdunski M (2006). Alpha-linolenic acid and riluzole treatment confer cerebral protection and improve survival after focal brain ischemia.. Neuroscience.

[24] Gruss M, Bushell TJ, Bright DP, Lieb WR, Mathie A (2004). Two-pore-domain K+ channels are a novel target for the anesthetic gases xenon, nitrous oxide, and cyclopropane.. Mol Pharmacol.

[25] Heurteaux C, Lucas G, Guy N, El Yacoubi M, Thümmler S (2006). Deletion of TREK-1, a background potassium channel, results in a depression-resistant phenotype.. Nature Neurosci.

[26] Sandoz G, Bell SC, Isacoff EY (2011). Optical probing of a dynamic membrane interaction that regulates the TREK1 channel.. Proc Natl Acad Sci U S A.

[27] Li ZB, Zhang HX, Li LL, Wang XL (2005). Enhanced expressions of arachidonic acid-sensitive tandem-pore domain potassium channels in rat experimental acute cerebral ischemia.. Biochem Biophys Res Commun.

[28] Mazella J, Petrault O, Lucas G, Deval E, Beraud-Dufour S (2010). Spadin, a sortilin-derived peptide, targeting rodent TREK-1 channels: a new concept in the antidepressant drug design.. PLoS Biol.

[29] Franks NP, Honore E (2004). The TREK K2P channels and their role in general anaesthesia and neuroprotection.. Trends Pharmacol Sci.

[30] Moha ou Maati H, Veyssiere J, Labbal F, Coppola T, Gandin C (2011). Spadin as a new antidepressant: absence of TREK-1-related side effects.. Neuropharmacology.

[31] Maingret F, Patel AJ, Lesage F, Lazdunski M, Honore E (1999). Mechano- or acid stimulation, two interactive modes of activation of the TREK-1 potassium channel.. J Biol Chem.

[32] Patel A, Lazdunski M, Honore E (2001). Lipid and mechano-gated 2P domain K(+) channels.. Curr Opin Cell Biol.

[33] Duprat F, Lesage F, Patel AJ, Fink M, Romey G (2000). The Neuroprotective Agent Riluzole Activates the Two P-Domain K^+^ Channels TREK-1 and TRAAK.. Mol Pharmacol.

[34] Lang-Lazdunski L, Heurteaux C, Vaillant N, Widmann C, Lazdunski M (1999). Riluzole prevents ischemic spinal cord injury caused by aortic crossclamping.. J Thorac Cardiovasc Surg.

[35] Malgouris C, Bardot F, Daniel M, Pellis F, Rataud J (1989). Riluzole, a novel antiglutamate prevents memory loss and hippocampal neuronal damage in ischemic gerbils.. J Neurosci.

[36] Goldberg MP, Choi DW (1993). Combined oxygen and glucose deprivation in cortical cell culture: calcium-dependent and calcium-independent mechanisms of neuronal injury.. J Neurosci.

[37] Tauskela JS, Brunette E, O'Reilly N, Mealing G, Comas T (2005). An alternative Ca2+-dependent mechanism of neuroprotection by the metalloporphyrin class of superoxide dismutase mimetics.. FASEB J.

[38] Cohen A, Sagron R, Somech E, Segal-Hayoun Y, Zilberberg N (2009). Pain-associated signals, acidosis and lysophosphatidic acid, modulate the neuronal K(2P)2.1 channel.. Mol Cell Neurosci.

[39] Finlayson K, Witchel HJ, McCulloch J, Sharkey J (2004). Acquired QT interval prolongation and HERG: implications for drug discovery and development.. Eur J Pharmacol.

[40] Kang J, Cheng H, Ji J, Incardona J, Rampe D (2010). In vitro electrocardiographic and cardiac ion channel effects of (-)-epigallocatechin-3-gallate, the main catechin of green tea.. J Pharmacol Exp Ther.

[41] Ren XQ, Liu GX, Organ-Darling LE, Zheng R, Roder K (2010). Pore mutants of HERG and KvLQT1 downregulate the reciprocal currents in stable cell lines.. Am J Physiol Heart Circ Physiol.

[42] Ducroq J, Moha ou Maati H, Guilbot S, Dilly S, Laemmel E (2010). Dexrazoxane protects the heart from acute doxorubicin-induced QT prolongation: a key role for I(Ks).. Br J Pharmacol.

[43] Buckler KJ, Honore E (2005). The lipid-activated two-pore domain K+ channel TREK-1 is resistant to hypoxia: implication for ischaemic neuroprotection.. J Physiol.

[44] Caley AJ, Gruss M, Franks NP (2005). The effects of hypoxia on the modulation of human TREK-1 potassium channels.. J Physiol.

[45] Patel AJ, Honore E, Maingret F, Lesage F, Fink M (1998). A mammalian two pore domain mechano-gated S-like K+ channel.. EMBO J.

[46] Blondeau N, Lauritzen I, Widmann C, Lazdunski M, Heurteaux C (2002). A potent protective role of lysophospholipids against global cerebral ischemia and glutamate excitotoxicity in neuronal cultures.. J Cereb Blood Flow Metab.

[47] Blondeau N, Widmann C, Lazdunski M, Heurteaux C (2002). Polyunsaturated fatty acids induce ischemic and epileptic tolerance.. Neuroscience.

[48] Lang-Lazdunski L, Blondeau N, Jarretou G, Lazdunski M, Heurteaux C (2003). Linolenic acid prevents neuronal cell death and paraplegia after transient spinal cord ischemia in rats.. J Vasc Surg.

[49] Pratt J, Rataud J, Bardot F, Roux M, Blanchard JC (1992). Neuroprotective actions of riluzole in rodent models of global and focal cerebral ischaemia.. Neurosci Lett.

[50] Wahl F, Allix M, Plotkine M, Boulu RG (1993). Effect of riluzole on focal cerebral ischemia in rats.. Eur J Pharmacol.

